# Thermodynamic Solubility Profile of Temozolomide in Different Commonly Used Pharmaceutical Solvents

**DOI:** 10.3390/molecules27041437

**Published:** 2022-02-21

**Authors:** Abdul Ahad, Faiyaz Shakeel, Mohammad Raish, Ajaz Ahmad, Yousef A. Bin Jardan, Fahad I. Al-Jenoobi, Abdullah M. Al-Mohizea

**Affiliations:** 1 Department of Pharmaceutics, College of Pharmacy, King Saud University, Riyadh 11451, Saudi Arabia; fsahmad@ksu.edu.sa (F.S.); mraish@ksu.edu.sa (M.R.); ybinjardan@ksu.edu.sa (Y.A.B.J.); aljenobi@ksu.edu.sa (F.I.A.-J.); amohizea@ksu.edu.sa (A.M.A.-M.); 2 Department of Clinical Pharmacy, College of Pharmacy, King Saud University, Riyadh 11451, Saudi Arabia; aajaz@ksu.edu.sa

**Keywords:** Apelblat and Van’t Hoff models, solubility, solution thermodynamics, temozolomide

## Abstract

The solubility parameters, and solution thermodynamics of temozolomide (TMZ) in 10 frequently used solvents were examined at five different temperatures. The maximum mole fraction solubility of TMZ was ascertained in dimethyl sulfoxide (1.35 × 10^−2^), followed by that in polyethylene glycol-400 (3.32 × 10^−3^) > Transcutol^®^ (2.89 × 10^−3^) > ethylene glycol (1.64 × 10^−3^) > propylene glycol (1.47 × 10^−3^) > H_2_O (7.70 × 10^−4^) > ethyl acetate (5.44 × 10^−4^) > ethanol (1.80 × 10^−4^) > isopropyl alcohol (1.32 × 10^−4^) > 1-butanol (1.07 × 10^−4^) at 323.2 K. An analogous pattern was also observed for the other investigated temperatures. The quantitated TMZ solubility values were regressed using Apelblat and Van’t Hoff models and showed overall deviances of 0.96% and 1.33%, respectively. Apparent thermodynamic analysis indicated endothermic, spontaneous, and entropy-driven dissolution of TMZ in all solvents. TMZ solubility data may help to formulate dosage forms, recrystallize, purify, and extract/separate TMZ.

## 1. Introduction

**Figure 1:**
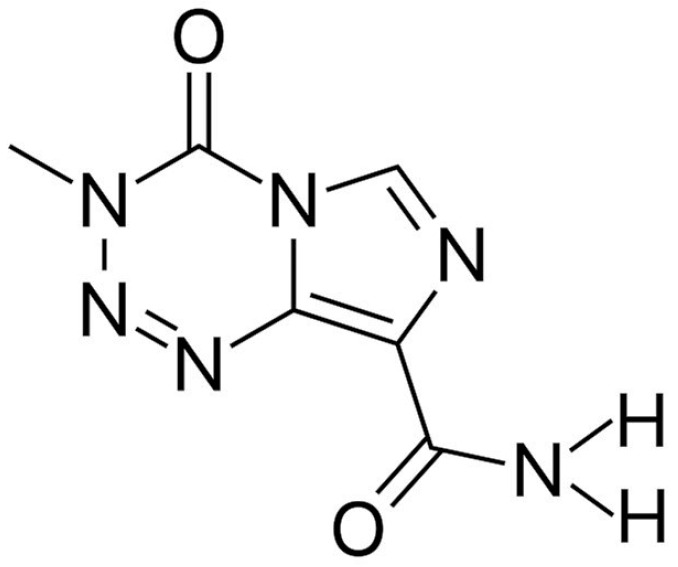
The chemical structure of temozolomide.

Temozolomide (TMZ; CAS number: 85622-93-1; molar mass: 194.15 g mol^−1^; molecular formula: C_6_H_6_N_6_O_2_; Figure 1) is indicated in brain tumors such as glioblastoma and malignant glioma as an oral adjuvant chemotherapy agent [1,2,3]. TMZ is usually prescribed by practitioners because of its comparatively exceptional cytotoxic characteristics for cancer cells [4]. TMZ is a prodrug and its metabolite alkylates nucleophiles (i.e., DNA) and induces cell death [5]. The speedy hydrolysis and poor solubility of the drug contributes to a lower biological half-life, and its deficient biodistribution restricts the anticancer activity through ordinary remedial treatment and results in non-specificity with an increased dose and multiple dosing [6,7].

Many approaches for the delivery of TMZ, including complexation [8], niosomes [6], solid lipid nanoparticles [9] lipid-based nanoparticles [1], nanomicelles [10], and chitosan engineered PAMAM dendrimers, [11] have previously been described for the augmentation of drug dissolution and bioavailability. The solubility of TMZ in H_2_O (2–4 mg/mL) and ethanol (EtOH, 0.4–0.6 mg/mL) is reported elsewhere [12]; however, the solubility of TMZ in solvents such as Transcutol^®^ (TC), propylene glycol (PG), isopropyl alcohol (IPA), ethylene glycol (EG), polyethylene glycol-400 (PEG-400), 1-butanol (1-BuOH), dimethyl sulfoxide (DMSO), and ethyl acetate (EA) has not been described thus far.

Thus, the objective of the current study was to determine the solubility of TMZ in 10 commonly used solvents (H_2_O, EtOH, IPA, EG, PG, PEG-400, TC, 1-BuOH, DMSO, and EA) at five different temperatures (298.2 K, 303.2 K, 308.2 K, 313.2 K, and 323.2 K) at atmospheric pressure. The studied temperature range from “*T* = 298.2 K to 323.15 K” and interval of 5.0 K or 10.0 K were selected randomly in such a way that the maximum evaluated temperature, i.e., “*T* = 323.2 K,” should not exceed the melting temperature of TMZ nor the boiling points of the studied solvents. The melting temperature of TMZ was determined to be 474.8 K using thermal analysis. The maximum investigated temperature, “*T* = 323.2 K, was much lower than the melting temperature of TMZ and the boiling temperatures of the studied solvents. As a result, the above temperature range was selected in this study. Solubility data were further utilized for apparent thermodynamic calculation using the equations described by Van’t Hoff and Krug et al. [13,14,15].

## 2. Materials and Methods

### 2.1. Materials

A list of materials and a table for materials is included in the Supplementary File (Table S1).

### 2.2. Methods

#### 2.2.1. TMZ Analysis

TMZ analysis was performed using a “HPLC system fitted with an SPD 20A UV/VIS detector (Shimadzu, Tokyo, Japan)” set at 330 nm. Methanol and 0.5% acetic acid (20:80, *v/v*) were pumped at a flow rate of 1 mL/min as the mobile phase. A “Nucleodur^®^ (C_18_, 5 μm, 150 × 4.6 mm; Macherey-Nagal, Düren, Germany)” HPLC column was used as the stationary phase and was maintained at room temperature [16].

#### 2.2.2. Assessment of TMZ by DSC, TGA, and PXRD

DSC, TGA, and PXRD analyses were carried out for the evaluation of the pure TMZ and equilibrated TMZ samples acquired from the bottom phase of the solubility sample in water [17,18]. DSC and TGA characterization of TMZ and its equilibrated sample from water were evaluated using the “DSC 8000 (Perkin Elmer, Shelton, CT, USA)” and “Pyris 1 TGA analyser (Perkin Elmer, Shelton, CT, USA)” respectively, in the temperature range from 323.2 K to 573.2 K. PXRD analysis was performed using the “Ultima IV Diffractometer (Rigaku Inc. Tokyo, Japan)”, and both TMZ and its equilibrated sample were analyzed from the 3° to 80° 2-theta range.

#### 2.2.3. Assessment of Solubility of TMZ

The static equilibrium method was utilized for the assessment of TMZ mole fraction solubility in commonly used solvents, namely, “H_2_O, EtOH, IPA, EG, PG, PEG-400, TC, 1-BuOH, DMSO, and EA” at five different temperatures, ranging from “298.2 K to 323.2 K” [19]. In the experiment, TMZ was added in surplus to known measures of each solvent; the mixture was vortexed strongly, and the samples were moved to a shaking water bath and maintained at 100 rpm for 72 h. Subsequently, every sample was drawn from the shaker bath and kept undisturbed for 1 day so that all the floating drug particles settled at the bottom of the vials [13,14]. Then, the supernatant from each sample was carefully removed and analyzed for drug content using the HPLC method [16]. Next, the TMZ solubility (*x*_e_) in the mole fraction in each solvent was assessed using Equation (1) [20]:

(1) $$\;x_{e}=\frac{m_{1}/M_{1}}{m_{1}/M_{1}+m_{2}/M_{2}}$$

where *m*_1_ and *m*_2_ are the masses of pure TMZ and the solvent (g) and *M*_1_ and *M*_2_ are the molar masses of TMZ and the solvent (g mol^−1^), respectively.

## 3. Results and Discussion

### 3.1. DSC, TGA, and PXRD of TMZ

**Figure 2:**
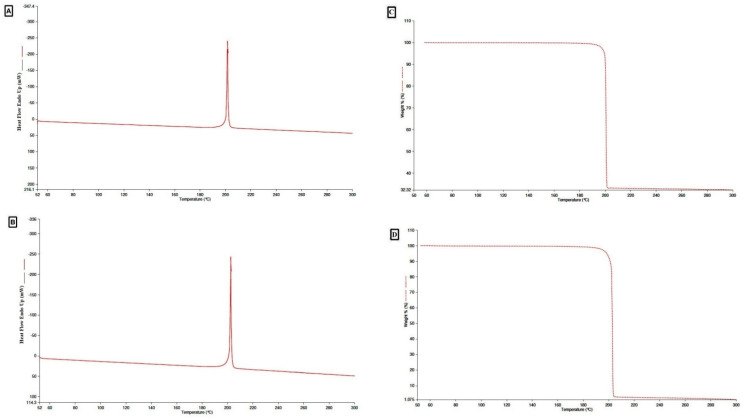
DSC curve of (**A**) TMZ, (**B**) TMZ equilibrated sample retrieved from H_2_O and TGA curve of (**C**) TMZ, (**D**) TMZ equilibrated sample retrieved from H_2_O.

The probable translation of TMZ after equilibrium can be identified by evaluating the solid state of TMZ in its pure and equilibrated forms. In DSC analysis, sharp exothermic signals were observed for TMZ and the equilibrated sample at the fusion temperatures (*T*_fus_) of 474.8 K and 475.7 K, respectively. A fusion enthalpy (∆*H*_fus_) of −107.05 kJ mol^−1^ and a ∆*H*_fus_ value of −110.85 kJ mol^−1^ were observed for the pure and equilibrated TMZ samples, respectively (Figure 2A,B). 

The *T*_fus_ and ∆*H*_fus_ for both pure TMZ and the equilibrated sample corresponded with each other, reflecting that TMZ was in the pure crystalline form [8] without any alteration to the amorphous or polymorphic form after equilibrium. The *T*_fus_ of 474.8 K for pure TMZ detected in the current study is consistent with the previously reported value of 482.2 K [8,21].

**Figure 3:**
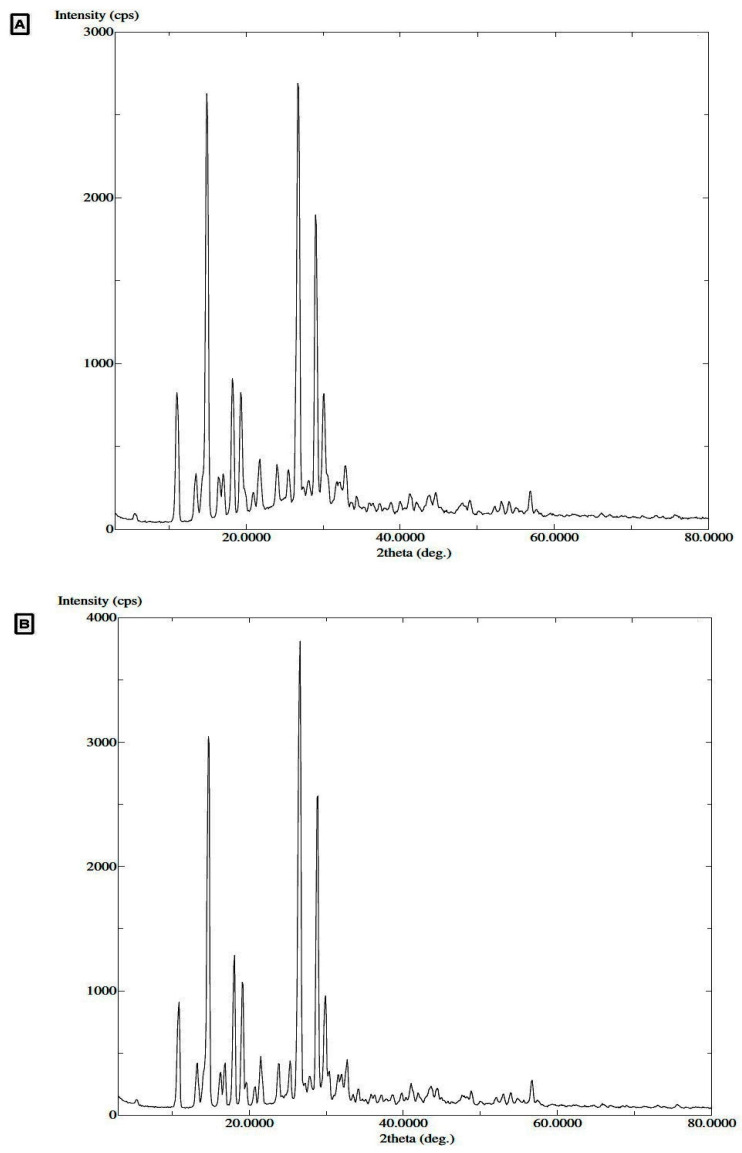
PXRD spectra of (**A**) TMZ, (**B**) TMZ equilibrated sample retrieved from H_2_O.

In addition, the DSC analysis results were further confirmed by the TGA analysis data. Pure TMZ and the equilibrated sample exhibited mass loss at approximately 473.13 K and 475.22 K, respectively (Figure 2C,D). Results for both the samples demonstrated that TGA signals for pure TMZ and the equilibrated sample were analogous to each other. The above results were further supported by the PXRD spectra; both the pure TMZ and the equilibrated sample showed analogous specific signals at various 2-theta values revealing the crystalline structure of pure TMZ and the equilibrated sample of TMZ from water (Figure 3A,B).

### 3.2. Experimental Solubility of TMZ

The *x*_e_ values of TMZ solubility in the 10 different investigated solvents are presented in Table 1. The data revealed that the maximum mole fraction solubility of TMZ was ascertained in DMSO (1.35 × 10^−2^), > PEG-400 (3.32 × 10^−3^), > TC (2.89 × 10^−3^), > EG (1.64 × 10^−3^), > PG (1.47 × 10^−3^), > H_2_O (7.70 × 10^−4^), > EA (5.44 × 10^−4^), > EtOH (1.80 × 10^−4^), > IPA (1.32 × 10^−4^), > 1-BuOH (1.07 × 10^−4^) at *T* = 323.2 K (Table 1).

The mole fraction solubility of TMZ was noticeably more prominent in DMSO, PEG-400, TC, EG, PG, H_2_O, and EA than in the other solvents considered. The *x*_e_ values of TMZ were much higher in PG than its *x*_e_ values in IPA. The Hansen solubility parameter (δ) of TMZ (30.30 MPa^1/2^) was closer to the δ value of PG (29.20 MPa^1/2^) than to the Hansen solubility parameter of IPA (22.30 MPa^1/2^). As a result, the *x*_e_ values of TMZ were much higher in PG than in IPA. The *x*_e_ values of TMZ were higher in H_2_O than in EtOH owing to the lower polarity of ethanol [22]. Thus, TMZ was greatly soluble in DMSO; soluble in PEG-400, TC, EG, and PG; soluble to some extent in H_2_O, EA, and EtOH; and poorly soluble in IPA and 1-BuOH.

### 3.3. Assessment of the Hansen Solubility Parameter

The assessment of the Hansen solubility parameter (δ) for TMZ and the other investigated solvents was calculated utilizing Equation (2) [23,24,25]:

(2) $$\delta^{2\;}=\delta_{d}^{2}+\delta_{p}^{2}+\delta_{h}^{2}$$

where δ_d_, δ_p_, and δ_h_ symbolize dispersion, polarity, and hydrogen-bonding parameters for Hansen solubility, respectively.

“HSPiP (version 4.1.07, Louisville, KY, USA)” was utilized to determine the values of these parameters (δ, δ_d_, δ_p_, and δ_h_) (Table S2). The TMZ δ value has not been described elsewhere. Nevertheless, the Hildebrand solubility parameter (δ_1_) for several solvents has been described [26]. The comparison between δ and δ_1_ is shown in Table S2. The calculated δ values for the analyzed solvents, for instance “H_2_O, EtOH, EG, PG, TC, IPA, 1-BuOH, and EA”, were found to be considerably near the stated δ_1_ values. However, the calculated δ values for PEG-400 and DMSO differed to some extent from the described δ_1_ values [26]. The δ value for TMZ was calculated as 30.30 MPa^1/2^, indicating that TMZ possesses moderate polarity (Table S2). The δ value for TMZ in DMSO was calculated as 23.60 MPa^1/2^. Moreover, the *δ* value of TMZ was found to be closer to those of pure EG (δ value 31.60) and PG (δ value 29.20), indicating the maximum solubility of TMZ in these solvents, as per the Hansen solubility parameter. 

### 3.4. Correlation of the x_e_ Values of TMZ

The TMZ *x*_e_ values were correlated using Apelblat and Van’t Hoff models. Equation (3) was employed to calculate the Apelblat model solubility (*x*^Apl^) values of TMZ [27,28]:

(3) $$\ln\;x^{\mathrm{Apl}}=A+\frac{B}{T}+C\ln\left(T\right)$$

where Apelblat model factors represented by *A*, *B*, and *C* were assessed using nonlinear multivariate regression analysis of the *x*_e_ data of TMZ presented in Table 1 [29].

**Figure 4:**
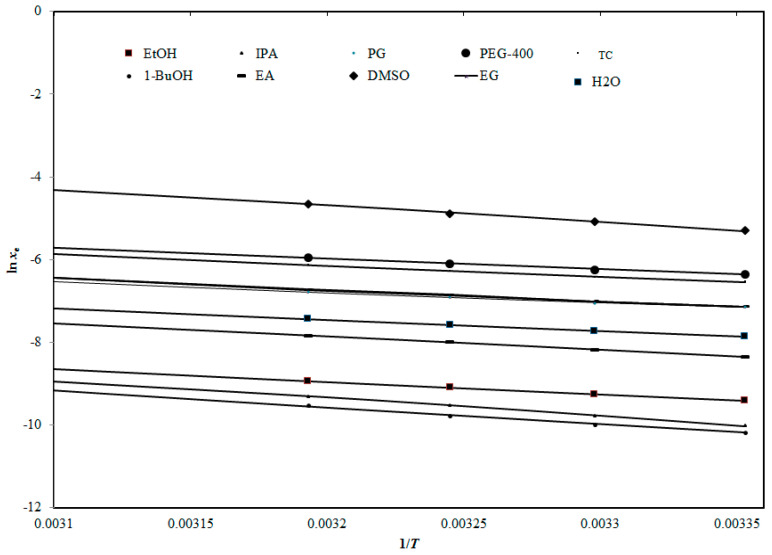
Correlation of ln *x*_e_ values of TMZ with “Apelblat model” in every solvent examined in the current study as a function of 1/*T*; symbols represent the experimental solubilities of TMZ and solid lines represent the solubilities of TMZ estimated by “Apelblat model”.

The TMZ *x*_e_ values were interconnected with the TMZ *x*^Apl^ values and evaluated in terms of root mean square deviations (*RMSD*) as well as *R*^2^ values. The *RMSD* for TMZ was estimated using Equation (4) [29]:

(4) $$RMSD={\left[\frac{1}{N}\overset{N}{\underset{i=1}{\sum }}{\left(\frac{x^{\mathrm{Apl}}-x_{e}}{x_{e}}\right)}^{2}\right]}^{\frac{1}{2}}$$

where *N* is the number of experimental temperature points. The graphical correlation between the logarithm *x*_e_ (ln *x*_e_) and ln *x*^Apl^ values of TMZ in each solvent as a function of 1/*T* is shown in Figure 4, revealing the interrelation between the ln *x*_e_ and ln *x*^Apl^ TMZ values in every examined solvent.

The maximal *RMSD* value for TMZ was observed for 1-BuOH (1.38%) and the lowest was for ethanol (0.22%) (Table 2). Furthermore, the *R*^2^ values of TMZ in the investigated solvents were observed to be in the range of 0.9973–0.9999 (Table 2). The *RMSD* and *R*^2^ values revealed a good correlation of the *x*_e_ values of TMZ with the Apelblat model.

Equation (5) was employed to calculate the Van’t Hoff model solubility (*x*^Van’t^) of TMZ [22].

(5) $$\ln\;x^{\mathrm{Van}’t}=a+\frac{b}{T}$$

where *a* and *b* are the model parameters of the Van’t Hoff model that are described by the schematization of the values of ln *x_e_* of TMZ as a function of 1/*T*. The *x*_e_ values of TMZ were interrelated using the *x*^Van’t^ values of TMZ in terms of *RMSD* and *R*^2^ values, and the relation between the ln *x*_e_ and ln *x*^Van’t^ values of TMZ in each solvent was calculated as a function of 1/*T* (Figure S1).

The data of Van’t Hoff correlation are shown in Table 3. The maximal *RMSD* value for TMZ was observed for IPA (1.90%) and the lowest was for EtOH (0.48%). The *R*^2^ values for TMZ in the examined solvents were in the range of 0.9957–0.9996. The assessed *RMSD* and *R*^2^ values indicated a good correlation for the *x*_e_ values of TMZ by the Van’t Hoff model.

### 3.5. Apparent Thermodynamic Analysis

The dissolution feature of TMZ in the examined solvents was evaluated using the experimental solubility data of TMZ in apparent thermodynamic analysis. For this analysis, different apparent standard thermodynamic parameters, for instance, Δ_sol_*H*^0^ (the apparent standard dissolution enthalpy), Δ_sol_*G*^0^ (the apparent standard Gibbs free energy), and Δ_sol_*S*^0^ (the apparent standard dissolution entropy) for TMZ dissolution, were evaluated using the Van’t Hoff and Krug et al. analysis methods [30]. The Δ_sol_*H*^0^ values for TMZ dissolution in the examined solvents were projected at the mean harmonic temperature (*T*_hm_) of 308.96 K by implementing Van’t Hoff analysis as described in Equation (6) [31,32]:

(6) (∂ln xe∂(1T−1Thm))P=−ΔsolH0R

The Van’t Hoff plots for the dissolution characteristic of TMZ in the examined solvents were in the form of linear plots, representing *R*^2^ values in the range from 0.9955 to 0.9995 (Figure S2).

The Krug et al. analysis was performed at *T*_hm_ = 308.96 K to evaluate the Δ_sol_*G*^0^ values to determine the dissolution behavior of TMZ using Equation (7) [32].

(7) $$\Delta_{\mathrm{sol}}G^{0}=-RT_{\mathrm{hm}}\times \mathrm{intercept}$$

The intercept value for TMZ in each examined solvent was ascertained from the Van’t Hoff plots displayed in Figure S2.

Furthermore, the Van’t Hoff and Krug et al. analytical approaches (Equation (8)) were utilized to calculate the values of Δ_sol_*S*^0^, which reveal the dissolution characteristics of TMZ [30,31,32].

(8) $$S^{0}=\frac{\Delta_{\mathrm{sol}}H^{0}-\Delta_{\mathrm{sol}}G^{0}}{T_{\mathrm{hm}}}$$

The data of apparent thermodynamic analysis for TMZ dissolution are presented in Table 4. The Δ_sol_*H*^0^ values for TMZ dissolution in the examined solvents were found to be positive values and within the range from 20.55 kJ mol^−1^ to 35.04 kJ mol^−1^. The maximum Δ_sol_*H*^0^ value for TMZ dissolution was observed in IPA (35.04 kJ mol^−1^), followed by that in 1-BuOH, EtOH, DMSO, EA, EG, TC, H_2_O, and PEG-400, and the minimum was observed in PG (20.55 kJ mol^−1^).

Further, the Δ_sol_*G*^0^ values for TMZ dissolution in the examined solvents were also found to be positive values and within the range from 12.44 to 24.97 kJ mol^−1^. The maximum Δ_sol_*G*^0^ value for TMZ dissolution was detected in 1-BuOH (24.97 kJ mol^−1^) followed by that in IPA, EtOH, EA, H_2_O, PG, EG, TC, and PEG-400, and the minimum was observed in DMSO (12.44 kJ mol^−1^). The minimum Δ_sol_*G*^0^ value in DMSO could be on account of the maximum solubility of TMZ in DMSO. The lowermost value of Δ_sol_*G*^0^ in DMSO indicated that little energy is needed for the solubilization and dissolution of TMZ in DMSO. The results of the Δ_sol_*G*^0^ analysis for TMZ dissolution were found to be in good correspondence with the TMZ solubility values. The positive values of Δ_sol_*H*^0^ and Δ_sol_*G*^0^ suggest that TMZ showed endothermic and spontaneous dissolution characteristics in all the solvents examined in the current study [14,33].

The Δ_sol_*S*^0^ values for the TMZ dissolution characteristic in all examined solvents were also detected as positive values and within the range from 8.74 J mol^−1^ K^−1^ to 64.03 J mol^−1^ K^−1^. The positive Δ_sol_*S*^0^ values suggest an entropy-driven dissolution of TMZ in all solvents. In general, the TMZ dissolution was regarded as endothermic and entropy-driven in all solvents examined in the current study [13,14,25]. The overall analysis shows that the dissolution behavior of TMZ in general was found to be endothermic, spontaneous, and entropy-driven in all the solvents considered in the current study [14,33].

## 4. Conclusions

In the present study, the experimental solubility and solution thermodynamics of TMZ, a classic DNA methylating anticancer drug, were examined in 10 frequently used solvents at five different temperatures. The solubility of TMZ in all the examined solvents was considerably augmented with an increase in temperature. The maximal mole fraction solubility of TMZ was detected in DMSO, followed by that in “PEG-400, TC, EG, PG, H_2_O, EA, EtOH, IPA, and 1-BuOH”. In conclusion, TMZ is considered to be highly soluble in DMSO; soluble in PEG-400, TC, EG, and PG; slightly soluble in H_2_O, EA, and EtOH, and poorly soluble in IPA and 1-BuOH.

## Figures and Tables

**Table 1:** Experimental solubilities (*x*_e_) of TMZ in mole fraction in different solvents (*S*) at “*T* = 298.2 K to 323.2 K” and “*p* = 0.1 MPa” ^a^ (values in parentheses are standard deviations).

*S*	*x*_e_				
	*T* = 298.2 K	*T* = 303.2 K	*T* = 308.2 K	*T* = 313.2 K	*T* = 323.2 K
H_2_0	3.90 × 10^−4^ (0.03)	4.41 × 10^−4^ (0.04)	5.10 × 10^−4^ (0.02)	5.93 × 10^−4^ (0.05)	7.70 × 10^−4^ (0.04)
EtOH	8.19 × 10^−5^ (0.02)	9.61 × 10^−5^ (0.03)	1.13 × 10^−4^ (0.05)	1.32 × 10^−4^ (0.04)	1.80 × 10^−4^ (0.06)
IPA	4.46 × 10^−5^ (0.03)	5.70 × 10^−5^ (0.02)	7.27 × 10^−5^ (0.04)	9.13 × 10^−5^ (0.05)	1.32 × 10^−4^ (0.06)
EG	7.99 × 10^−4^ (0.05)	8.94 × 10^−4^ (0.06)	1.05 × 10^−3^ (0.05)	1.21 × 10^−3^ (0.07)	1.64 × 10^−3^ (0.05)
PG	7.83 × 10^−4^ (0.04)	8.61 × 10^−4^ (0.02)	9.98 × 10^−4^ (0.03)	1.14 × 10^−3^ (0.06)	1.47 × 10^−3^ (0.07)
PEG-400	1.73 × 10^−3^ (0.06)	1.93 × 10^−3^ (0.04)	2.26 × 10^−3^ (0.05)	2.60 × 10^−3^ (0.06)	3.32 × 10^−3^ (0.08)
TC	1.45 × 10^−3^ (0.05)	1.62 × 10^−3^ (0.04)	1.90 × 10^−3^ (0.06)	2.21 × 10^−3^ (0.05)	2.89 × 10^−3^ (0.06)
1-BuOH	3.82 × 10^−5^ (0.02)	4.58 × 10^−5^ (0.03)	5.73 × 10^−5^ (0.02)	7.25 × 10^−5^ (0.04)	1.07 × 10^−4^ (0.05)
EA	2.36 × 10^−4^ (0.04)	2.81 × 10^−4^ (0.05)	3.40 × 10^−4^ (0.04)	3.95 × 10^−4^ (0.03)	5.44 × 10^−4^ (0.02)
DMSO	4.97 × 10^−3^ (0.06)	6.20 × 10^−3^ (0.05)	7.59 × 10^−3^ (0.07)	9.57 × 10^−3^ (0.08)	1.35 × 10^−2^ (0.09)

^a^ The standard uncertainties *u* are *u*(*T*) = 0.20 K, *u*(*p*) = 0.003 MPa and *u*_r_(*x*_e_) = 1.60%.

**Table 2:** Results of Apelblat model in terms of model parameters (*A*, *B* and *C*), *R*^2^ and % *RMSD* values for TMZ in various solvents (*S*) (values in parentheses are standard deviations).

*S*	*A*	*B*	*C*	*R* ^2^	*RMSD* (%)	Overall *RMSD* (%)
H_2_O	−96.198 (2.2100)	1822.4 (11.123)	14.432 (0.82120)	0.9990	0.74	
EtOH	−117.90 (2.3210)	2428.3 (14.421)	17.613 (0.92120)	0.9999	0.22	
PG	−252.63 (3.5301)	9229.5 (42.630)	37.651 (1.1802)	0.9983	1.16	
PEG-400	−60.439 (1.8203)	327.96 (6.6130)	9.2970 (0.7150)	0.9973	1.19	
TC	−174.04 (3.3420)	5427.5 (32.411)	26.202 (1.0501)	0.9983	1.17	0.96
EG	−334.82 (4.6401)	12741 (64.402)	50.012 (1.9801)	0.9991	0.99	
IPA	408.58 (6.7104)	−22884 (91.420)	−60.002 (2.0201)	0.9997	0.93	
1-BuOH	−132.74 (3.2308)	2236.8 (13.802)	20.194 (1.0020)	0.9984	1.38	
EA	59.685 (1.3002)	−5864.4 (36.420)	−8.4900 (0.56020)	0.9994	0.62	
DMSO	255.77 (3.8502)	−15330 (72.410)	−36.800 (1.9200)	0.9993	1.20	

**Table 3:** Results of Van’t Hoff model in terms of model parameters (*a* and *b*), *R*^2^ and % *RMSD* values for TMZ in various solvents (*S*) (values in parentheses are standard deviations).

*S*	*a*	*b*	*R* ^2^	*RMSD* (%)	Overall *RMSD* (%)
H_2_0	1.0626 (0.04010)	−2661.5 (16.322)	0.9986	0.92	
EtOH	0.79120 (0.02020)	−3044.1 (19.120)	0.9996	0.48	
PG	1.1262 (0.05000)	−2474.9 (13.410)	0.9958	1.54	
PEG-400	2.2140 (0.08105)	−2559.5 (14.830)	0.9972	1.28	
TC	2.5542 (0.09600)	−2716.4 (21.201)	0.9973	1.36	1.33
EG	2.2560 (0.09401)	−2806.6 (24.091)	0.9957	1.72	
IPA	4.1474 (0.21000)	−4219.7 (33.202)	0.9979	1.90	
1-BuOH	3.3438 (0.02200)	−4036.7 (31.403)	0.9981	1.61	
EA	2.4479 (0.18040)	−3219.8 (28.530)	0.9994	0.99	
DMSO	7.7170 (0.89401)	−3881.2 (31.640)	0.9985	1.52	

**Table 4:** Results of “apparent thermodynamic analysis” in terms of Δ_sol_*H*^0^, Δ_sol_*G*^0^, Δ_sol_*S*^0^ and *R*^2^ values for TMZ in various solvents (*S*) ^b^.

*S*	Δ_sol_*H*^0^/kJ mol^−1^	Δ_sol_*G*^0^/kJ mol^−1^	Δ_sol_*S*^0^/J mol^−1^ K^−1^	*R* ^2^
H_2_0	22.10 (0.7400)	19.39 (0.5801)	8.748 (0.1001)	0.9985
EtOH	32.76 (1.710)	23.17 (0.8001)	31.01 (1.020)	0.9995
PG	20.55 (0.6501)	17.68 (0.3800)	9.279 (0.1200)	0.9957
PEG-400	21.55 (0.6700)	15.59 (0.3400)	18.32 (0.2500)	0.9971
TC	22.55 (0.7810)	16.02 (0.3600)	21.14 (0.5700)	0.9971
EG	23.30 (0.8100)	17.54 (0.3900)	18.66 (0.5200)	0.9955
IPA	35.04 (1.810)	24.43 (0.91703)	34.35 (0.9202)	0.9980
1-BuOH	33.52 (1.710)	24.97 (0.8800)	27.66 (0.8100)	0.9980
EA	26.74 (0.9300)	20.48 (0.7501	20.25 (0.6400)	0.9994
DMSO	32.23 (1.410)	12.44 (0.2200)	64.03 (1.920)	0.9985

^b^ The relative uncertainties are *u*(Δ_sol_*H*^0^) = 0.21 kJ mol^−1^, *u*(Δ_sol_*G*^0^) = 0.21 kJ mol^−1^ and *u*(Δ_sol_*S*^0^) = 0.62 J mol^−1^ K^−1.^

## Data Availability

The data generated from the experiments have been presented in the results.

## Supplementary Materials

The following are available online, Material list, Figure S1: Correlation of ln xe values of TMZ with “Van’t Hoff model” in various neat solvents as a function of 1/T, Figure S2: Van’t Hoff plots for TMZ plotted between ln xe and 1/T-1/Thm in various examined solvents, Table S1: A sample table for materials and Table S2: Hansen solubility parameters of TMZ and various neat solvents at T = 298.2 K.

## Sample Availability

Sample of the compound TMZ is available from the authors.
