# Supplementary material for: Thermodynamic Solubility Profile of Temozolomide in Different Commonly Used Pharmaceutical Solvents
Source: Molecules. 2022 Feb 21;27(4):1437. doi: 10.3390/molecules27041437 (PMC8879539; doi:10.3390/molecules27041437)
Supplement: Supplementary file 1 [file molecules-27-01437-s001.zip › molecules-1545998-supplementary.pdf]

Thermodynamic solubility profile of temozolomide in different commonly used pharmaceutical solvents

Abdul Ahad[a,*], Faiyaz Shakeel[a], Mohammad Raish[a], Ajaz Ahmad[b], Yousef A. Bin Jardan[a], Fahad I. Al-Jenoobi[a], Abdullah M. Al-Mohizea[a]

[a]"*Department of Pharmaceutics, College of Pharmacy, King Saud University, Riyadh 11451, Saudi Arabia*".

[b]"*Department of Clinical Pharmacy, College of Pharmacy, King Saud University, Riyadh 11451, Saudi Arabia*".

***Corresponding Author:* Dr. Abdul Ahad**

"Department of Pharmaceutics, College of Pharmacy,

King Saud University, P.O. Box 2457, Riyadh 11451, Saudi Arabia".

Email: abdulahad20@yahoo.com, aahad@ksu.edu.sa

Materials

TMZ was purchased from “Tokyo Chemical Industry Co., Ltd. (Tokyo, Japan)”. DMSO and 1-BuOH were bought from “BDH Laboratory Supplies (Poole, UK)”. EA was acquired from “Winlab Ltd. (Leicestershire, UK)”. EG and PG were obtained from “Anova Chem (Hurden, Switzerland)”. EtOH was bought from “Sigma-Aldrich (St. Louis, MO, USA)”. IPA was obtained from “Panreac Química, S.A.U. (Barcelona, Spain)”. Milli-Q H_2O was obtained from a “Millipore system (Millipore SAS, Molsheim, France)”. PEG-400 was obtained from “Merck Schuchardt (Munchen, Germany)”. TC was purchased from “Sigma-Aldrich (St. Louis, MO, USA)” (Table S1).

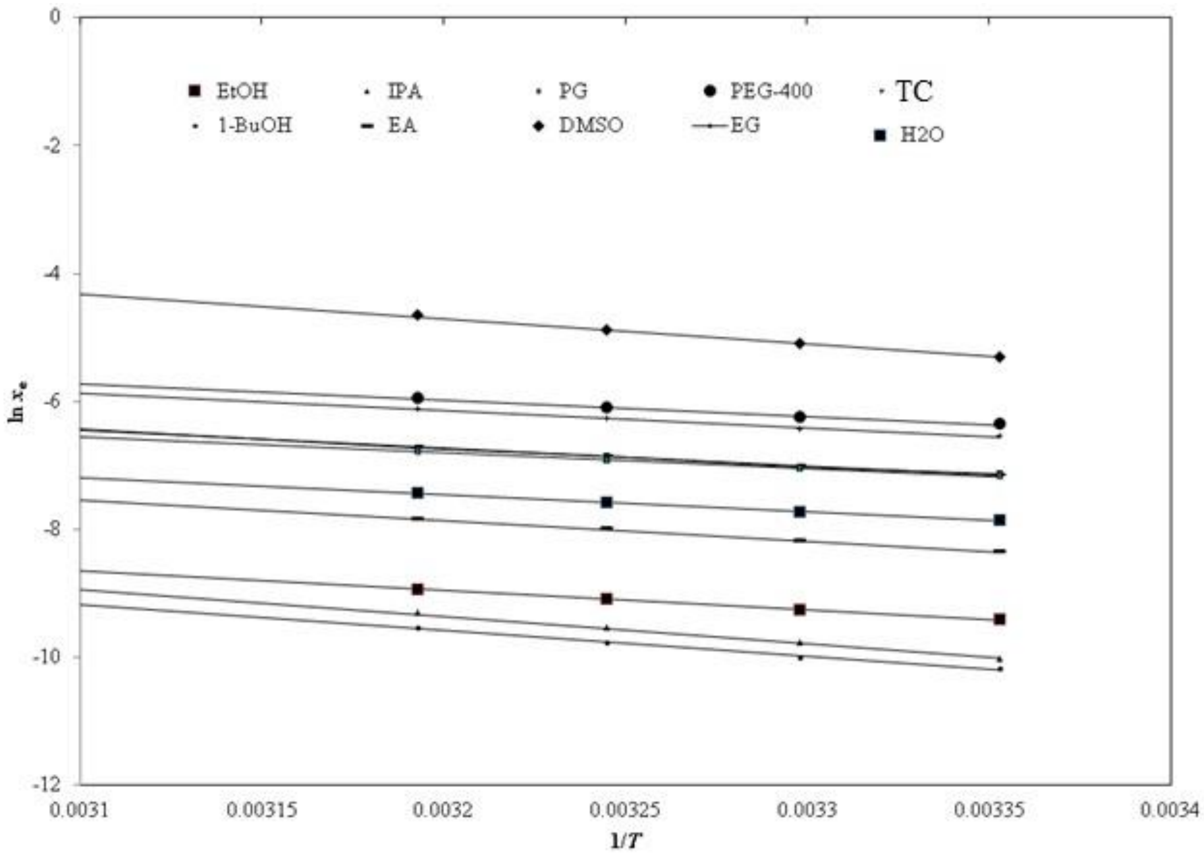

Figure S1: Correlation of ln x_e values of TMZ with "Van't Hoff model" in various neat solvents as a function of $1/T$; symbols represent the experimental solubilities of TMZ and solid lines represent the solubilities of TMZ estimated by "Van't Hoff model".

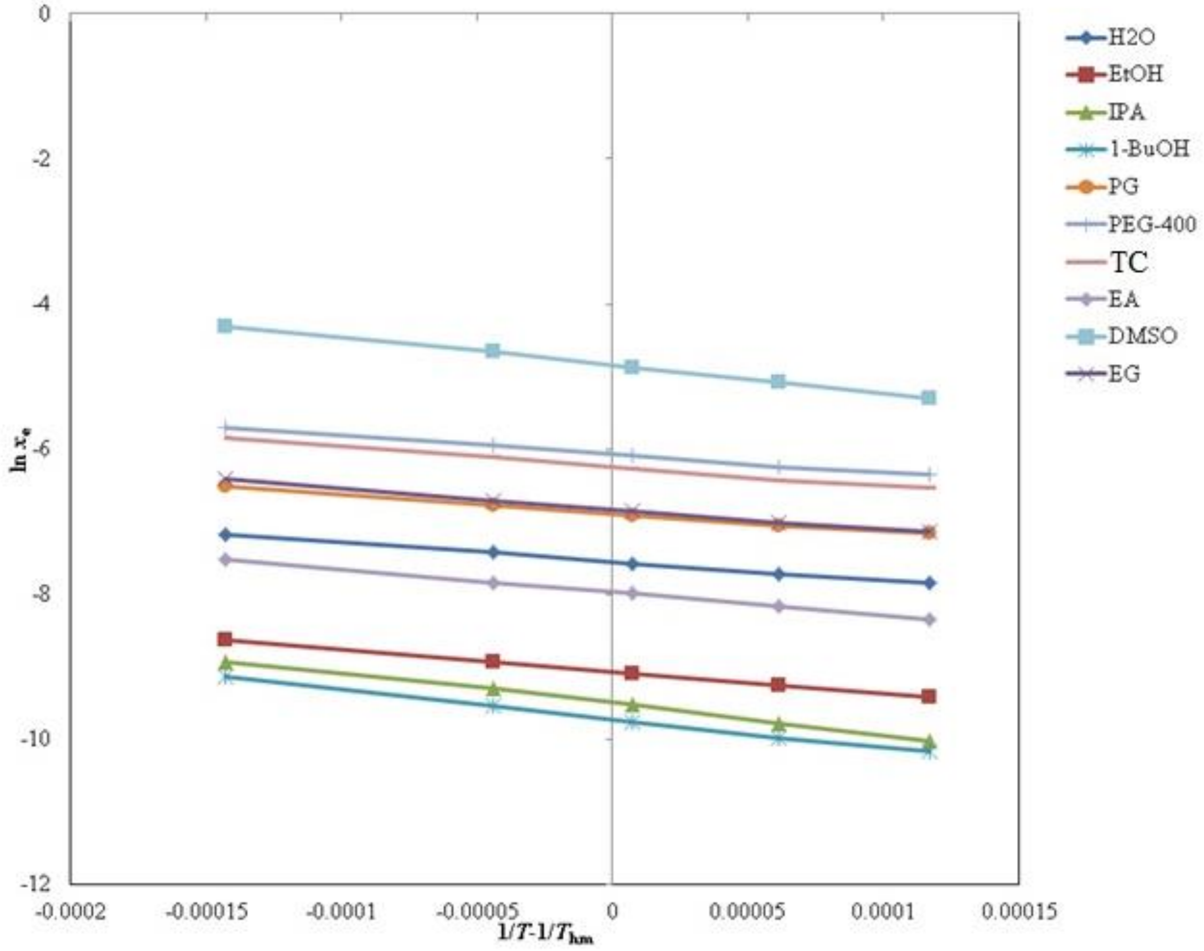

Figure S2: Van’t Hoff plots for TMZ plotted between ln x_e and $1/T$-$1/T_{hm}$ in various examined solvents.

Table S1. A sample table for materials.

Material	Molecular formula	Molar mass (g mol^{-1})	CAS Registry no.	Purification method	Mass fraction purity	Source
TMZ	$C_6H_6N_6O_2$	194.15	85622-93-1	None	0.980	Tokyo Chemical Industry
EtOH	C_2H_5OH	46.07	64-17-5	None	0.995	Sigma-Aldrich
EG	$C_2H_6O_2$	62.07	107-21-1	None	0.996	Annova Chem
TC	$C_6H_{14}O_3$	134.17	111-90-0	None	0.999	Gattefosse
PG	$C_3H_8O_2$	76.09	57-55-6	None	0.995	Annova Chem
PEG-400	$H(OCH_2CH_2)_nOH$	400	25322-68-3	None	0.999	Merck Schuchardt
IPA	C_3H_8O	60.10	67-63-0	None	0.997	Panreac Química
1-BuOH	$C_4H_{10}O$	74.12	71-36-3	None	0.998	BDH Laboratory

						Supplies
DMSO	C_2H_6OS	78.13	67-68-5	None	0.990	BDH Laboratory Supplies
EA	$C_4H_8O_2$	88.11	141-78-6	None	0.998	Winlab limited
H_2O	H_2O	18.07	7732-18-5	None	-	Milli-Q

Table S2. Hansen solubility parameters of TMZ and various neat solvents at T = 298.2 K.

Components	Hansen solubility parameters			
	δ_d/MPa$^{1/2}$	δ_p/MPa$^{1/2}$	δ_h/MPa$^{1/2}$	δ/MPa$^{1/2}$
TMZ	17.80	20.50	13.40	30.30
H_2O	15.50	16.00	42.30	47.80
EtOH	16.20	8.40	17.60	25.40
EG	18.00	11.10	23.40	31.60
PG	17.40	9.10	21.70	29.20
PEG-400	14.60	7.50	9.40	18.90
TC	16.30	7.20	11.90	21.40
IPA	15.80	6.60	14.30	22.30
1-BuOH	15.90	6.30	15.20	22.90
EA	15.70	5.60	7.00	18.10
DMSO	17.40	14.20	7.30	23.60
